# Parkinson’s disease LRRK2 mutations dysregulate iron homeostasis and promote oxidative stress and ferroptosis in human neurons and astrocytes

**DOI:** 10.1101/2025.09.26.678370

**Published:** 2025-09-28

**Authors:** Adamantios Mamais, Richard D. Batchelor, Aravindraja Chairmandurai, Nitya Subrahmanian, Nunziata Maio, Christopher D. Vulpe, Matthew J. LaVoie

**Affiliations:** 1. Center for Translational Research in Neurodegenerative Disease, Fixel Institute for Neurologic Diseases, Department of Neurology, University of Florida, Gainesville, FL, USA.; 2. *Eunice Kennedy Shriver* National Institute of Child Health and Human Development, NIH, Bethesda, MD, USA.; 3. Center for Environmental and Human Toxicology, Department of Physiological Sciences, College of Veterinary Medicine, University of Florida, Gainesville, FL, USA.

**Keywords:** LRRK2, Parkinson’s disease, iron, lysosomes, ferroptosis, Rab8a, iPSC, neurons, astrocytes

## Abstract

**Background:**

Iron accumulation is a hallmark of sporadic and familial Parkinson’s disease (PD) pathology and correlates with clinical motor symptom severity. The biochemical mechanisms driving iron dyshomeostasis in PD brain and whether these are early or late event in the neurodegenerative process remain unknown. Elevated nigral iron levels have been reported in LRRK2 mutation carriers, both in PD patients compared to idiopathic PD and in asymptomatic carriers relative to controls, suggesting that iron accumulation precedes clinical onset in LRRK2-associated PD. However, the precise consequence of pathogenic LRRK2 mutations on cellular iron handling within neurons and glial cells remains unclear.

**Methods:**

Here, we investigated different readouts of iron homeostasis in iPSCs and iPSC-derived neurons and astrocytes from PD patients harboring G2019S or R1441C/G LRRK2 mutations or healthy controls, as well as an isogenic iPSC panel with the same variants. By using high-content and super-resolution microscopy of iron-specific probes, we assayed iron content and distribution in cells and examined the downstream effects of iron dyshomeostasis on ferroptosis signaling.

**Results:**

We show that heterozygous LRRK2 mutations dysregulate cellular iron across iPSCs, neurons and astrocytes, in a kinase-dependent manner. Lysosomal ferrous iron storage was consistently elevated across iPSCs, iPSC-derived neurons and astrocytes carrying LRRK2 mutations. LRRK2 regulates Rab GTPase function through their direct phosphorylation, and our prior work revealed significant but divergent lysosomal phenotypes between Rab8a and Rab10 knockout models. Here, we report that Rab8a knockout recapitulates key aspects of LRRK2 mutation phenotypes on intracellular iron and ferritin levels, although with differences in magnitude and specificity. By contrast, Rab10 deficiency showed opposing effects, suggesting distinct roles for these well-established LRRK2 substrates in iron homeostasis. Finally, we show that basal lipid peroxidation and ROS levels are elevated in isogenic LRRK2 mutant neurons, while iron chelation was sufficient to reduce LRRK2-dependent ROS.

**Conclusions:**

Together, our findings demonstrate that LRRK2 mutations disrupt iron homeostasis across disease-relevant cell types and establish a discrete biochemical pathway linking LRRK2 signaling and vulnerability to ferroptosis. Ongoing work aims to further dissect the roles of Rab substrates in these pathways.

## Background

Iron accumulation in the substantia nigra is a well-recognized pathological hallmark of Parkinson’s disease (PD), observed in both sporadic and familial forms of the disorder and in other neurodegenerative conditions^1–7^. This iron overload correlates with neuromelanin loss in post-mortem PD brains^8^ and is detectable *in vivo* through advanced MRI techniques, such as quantitative susceptibility mapping (QSM), where iron deposition in the substantia nigra pars compacta (SNc) has been correlated with motor symptom severity^9^. Notably, iron-sensitive imaging aids in distinguishing PD from other parkinsonian syndromes such as multiple system atrophy (MSA) and progressive supranuclear palsy (PSP)^10–13^. Despite decades of evidence linking iron dyshomeostasis to PD pathogenesis, the molecular underpinnings that drive brain iron accumulation and neuronal vulnerability remain incompletely understood.

Iron’s potential to promote oxidative stress through Fenton chemistry and the generation of reactive oxygen species (ROS) makes it a likely contributor to dopaminergic (DA) neuron degeneration^14,15^. Ferroptosis, a regulated form of iron-dependent cell death, has emerged as a key mechanism linking iron overload to neuronal loss^16–20^, but how genetic risk factors for PD intersect with ferroptotic pathways is not fully defined. Among these genetic factors, mutations in leucine-rich repeat kinase 2 (LRRK2), the most common cause of familial PD^21^, have been implicated in vesicular trafficking defects and lysosomal dysfunction^22–28^, processes that could plausibly disrupt iron handling. Indeed, clinical imaging studies reveal that individuals carrying pathogenic LRRK2 mutations, whether symptomatic or asymptomatic, exhibit elevated nigral iron levels compared to idiopathic PD patients and healthy controls^29^, suggesting that iron dyshomeostasis could be an early and important feature in LRRK2-associated PD. However, the cellular and molecular mechanisms underlying these observations remain unknown.

Autosomal dominant missense mutations in LRRK2 cause a late-onset form of familial PD while genome-wide association studies have identified risk-factor variants of LRRK2 linked to idiopathic PD^21^. PD-linked mutations segregate within the kinase domain (e.g. G2019S) and the Roc-COR tandem domain (e.g. R1441C, Y1699C) of LRRK2 and increase phosphorylation towards a subset of Rab GTPases including Rab8a and Rab10^30,31^ disrupting endolysosomal dynamics^22–25^. Given that Rab GTPases govern critical steps in membrane trafficking and receptor recycling^32^ these pathways offer a potential intersection between LRRK2 function and iron regulation. In our previous work we observed alterations in trafficking of the transferrin receptor (TfR), that mediates iron uptake in most cell types^33,34(p1),35(p13),36^, in mutant LRRK2 overexpression models^28^. Importantly, we reported increase in brain ferritin and iron load in homozygous G2019S KI mice compared to WT controls, in acute pro-inflammatory conditions^28^. Whether such disturbances occur across different LRRK2 mutations, particularly in human neurons and glia when expressed in the heterozygous state, remains unclear. Astrocytes are key regulators of brain iron levels and availability as well as being implicated in PD neuropathology^37–43^. While LRRK2 expression is enriched in astrocytes and we and others have reported effects of LRRK2 mutations on astrocytic function^44,45^, how endogenous LRRK2 mutations affect cellular iron status and distribution in human astrocytes and neurons as well as the role of altered iron in neuronal damage remain unexplored.

In this study, we leverage patient-derived and isogenic induced pluripotent stem cell (iPSC) models to investigate how pathogenic LRRK2 mutations influence iron regulation in human neurons and astrocytes. By examining a wide panel of iron-related phenotypes, we reveal convergent effects of heterozygous R1441C, Y1699C and G2019S LRRK2 mutations on intracellular and lysosomal iron content that were rescued by LRRK2 inhibition. Furthermore, we report distinct effects of LRRK2 mutations on ferritin and a concomitant effect on basal IRP activity, suggesting alterations in ferritin expression and regulation. Rab8a deficiency recapitulated key iron-related defects observed in LRRK2 mutant cells while Rab10 KO cells showed opposite phenotypes, consistent with work from us and others supporting divergent Rab8a/Rab10 biology. Lastly, LRRK2 mutant neurons showed ROS accumulation and lipid peroxidation that was reversed by iron chelation, supporting the engagement of the ferroptotic pathway. Our data highlight integral cellular iron regulation pathways that are dysregulated by LRRK2 mutations in human iPSC models, mechanistically link a direct LRRK2 kinase substrate to these effects, and suggest a central role for iron dyshomeostasis in the reported interplay between LRRK2 signaling and ROS in neurons.

## Methods

### Induced pluripotent stem cell (iPSCs) cultures

We used iPSC lines carrying heterozygous LRRK2 mutations from three independent sources. BR33 were derived from a Caucasian donor, who was deeply phenotyped as part of the ROS/MAP longitudinal aging studies^46,47^ and determined to not be cognitively impaired at death at age >89 and free from genetic variants that confer risk of PD. ND35371 (R1441C heterozygous) and ND50051 (G2019S heterozygous) were obtained by the NINDS Repository. Five G2019S heterozygous, two R1441G heterozygous and five WT iPSC lines were obtained from the Parkinson’s Progression Markers Initiative dataset (PPMI, ppmi-info.org) along with longitudinal clinical data. The isogenic mutant LRRK2 lines (all heterozygous) were a kind gift from Dr Mark Cookson (NIA, NIH)^48^.

### CRISPR/Cas9 Genome Editing of iPSCs and HEK293 cells

Rab8a and Rab10 knock out lines were generated in iPSCs and HEK293 cells as described before^49^. Briefly, single guide RNAs (sgRNAs) were selected using a web-based design tool (http://crispr.mit.edu). Rab8a sgRNA: 5’ GAACTGGATTCGCAACATTG 3’; Rab10 sgRNA: 5’ ATGGCTTAGAAACATAGATG 3’. These were cloned into pXPR_003 (Addgene #52963), modified to express the neomycin resistance gene instead of the puromycin resistance gene and sequenced using the primer 5′-GATACAAGGCTGTTAGAGAGATAATT-3′ to determine clones that successfully integrated the sgRNA. BR33 iPSCs were generated and characterized in collaboration with the New York Stem Cell Foundation (NYSCF) using described methods ^47,50,51^. iPSCs were co-transfected with plasmids that express dCas9 (Addgene 61425) and the sgRNA plasmid. After 2 days, cells that were successfully transfected with the two plasmids were selected by puromycin treatment for 4 days. Individual clones were identified by plating ∼1 cell per well in a 96-well dish and allowed to grow for 2 weeks. Monoclonal lines were expanded, sequenced and stocked. Amplification of Rab8a and Rab10 genes was conducted according to manufacturer’s protocol (Invitrogen K2030–01) and homozygous gene editing was confirmed by Sanger sequencing. Multiple sequence alignment was performed using ClustalW in BioEdit.

### iPSC differentiation into mature astrocytes

iPSCs were differentiated into iAs using the protocol developed by Canals and colleagues^52^. Briefly, iPSCs were maintained in mTeSR media and transduced with lentivirus to stably express pTet-O-NFIB (hygromycin), pTet-O-SOX9 (puromycin) and FUdelta GW-rtTA. Differentiation of pure human iA cultures was achieved by culturing transduced cells in doxycycline to induce expression of Sox9 and Nfib and to select against non-differentiated cells with selection media (puromycin and hygromycin). Human iAs were studied at day ~21 post-differentiation. iAs show marked expression of GFAP by staining and immunoblot and robust expression of LRRK2 and relevant Rab GTPases.

### iPSCs differentiation into cortical neurons

iPSCs were differentiated into mature cortical neurons, as before^49^Sanyal, et al, Lee, Feenster et al.. Briefly, iPSCs were cultured in StemFlex (A33493) media and co-transduced with lentivirus packaged with pTet-O-NGN2-puro and Fudelta GW-rtTA plasmids (Zhang et al., 2013) for 2 days and passaged for expansion. NGN2-transduced iPSCs were thawed in StemFlex media with ROCK inhibitor (10 μM; Stemcell Technologies, 72304) and plated at 2 × 106 cells/10 cm plate and grown until 75% confluent. For differentiation, on day 1 cells were fed with KnockOut media (Gibco 10829.018) supplemented with KnockOut Serum Replacement (Invitrogen 10928–028), 1% MEM non-essential amino acids (Invitrogen 11140), 1% GlutaMAX (Gibco 35050061) and 0.1% BME (Invitrogen 21985–023) (KSR) with doxycycline (2 μg/ml, Sigma, D9891–5g) to induce NGN2 expression. On day 2, they were fed with a 1:1 ratio of KSR:N2B media (DMEM F12 supplemented with 1% GlutaMAX, 3% dextrose and N2-Supplement B; StemCell Technologies 07156) with puromycin (5 μg/ml; Life Technologies, A11138–03) and doxycycline to select for transduced cells. On day 3, the cells were fed with N2B media with B27 (1:100; Life technologies, 17504–044), puromycin, and doxycycline. On day 4, induced neurons (iNs) were frozen down in 10% DMSO/FBS in Neurobasal media (NBM Gibco 21103–049) supplemented with B27, BDNF (Peprotech, 450–02), CNTF (Peprotech, 450–13), and GDNF (Peprotech, 450–10) all at 100 ng/uL, ROCK inhibitor (10 μM), puromycin, and doxycycline. iNs were plated and grown in NBM with B27, BDNF, CNTF, GDNF, puromycin, and doxycycline until day 21.

### PPMI UPDRSIII data

Data used in the preparation of this article was obtained from the Parkinson’s Progression Markers Initiative (PPMI) database (www.ppmi-info.org/access-data-specimens/download-data), RRID:SCR_006431. Openly available UPDRSIII data for five G2019S and two R1441G mutations carriers were used (Tier 1 data downloaded between 03 November 2023 and 10 November 2023).

### IRE-binding activity assays

IRP activity was assessed using biotinylated IRE RNA probes, as previously described^53^. Briefly, probes were 3’-biotinylated and sourced from Eurofins Genomics or IDT. Cell/tissue lysates were prepared in lysis buffer (40 mM KCl, 25 mM Tris-Cl pH 7.5, 1% Triton X-100) with protease inhibitors (ThermoFisher #78430), 1 mM DTT, and 20 U/ml RNase inhibitor (ThermoFisher #AM2696). For binding, 5 pmol of annealed IRE probes were incubated with 100 μg DynaBeads M280 for 20 min at room temp, washed three times, then mixed with 100 μg lysates for another 20 min. IRP-bound complexes were isolated magnetically, washed, and resuspended in 100 μL LDS buffer, denatured at 95 °C for 5 min, and analyzed by western blot (25 μL on 4–12% Bis-Tris gels) for IRP1 and IRP2.

### ICP-MS

iPSCs in culture were collected in cold PBS and divided equally into two tubes for ICP-MS and biochemical analysis of protein levels, pelleted and frozen in dry ice. iPSC frozen pellets were resuspended in 200 μl PBS and mixed with 200 μl of concentrated trace-metal-grade nitric acid (Fisher) in 15 ml Falcon tube. The samples were digested at 85°C overnight, diluted to 4 ml with deionized water, and then analyzed by Agilent 7900 ICP-MS at the Analytical Toxicology Core Laboratory (ATCL) at the University of Florida. Metal amounts were normalized to protein amounts for cell number.

### Cell Treatments

For iron overload or chelation, cells were treated with 150μM ferric ammonium citrate (FAC) (SIGMA) or 100μM of deferoxamine in normal media overnight prior to analysis by imaging or western blotting. For rescue experiments, cells were treated with 100nM MLi2 (Tocris) for 7 days in culture prior to analysis.

### Western Blot

Cells were lysed in cell lysis buffer (50 mM Tris-HCl, 150 mM NaCl, 0.5 mM EDTA, 0.5% (v/v) sodium deoxycholate, 1% (v/v) NP-40, pH 8) with protease and phosphatase inhibitors for 30 min. Lysates were centrifuged at 14,000g for 15 min at 4°C, and supernatants were quantified using the BCA assay. Samples were prepared in 1x SDS-PAGE loading buffer, denatured at 65°C for 10 min, resolved on an SDS-polyacrylamide gel, and transferred to PVDF membrane. Membranes were blocked with 5% BSA (Sigma) prior to probing with primary antibodies against Fth1 (Cell Signaling 4393S), Ftl (abcam AB69090), TfR1 (13–6800), DMT1 (abcam AB123085), Cyclophilin B (abcam AB16045). Secondary LiCOR antibodies were used for detection using the LiCOR model M scanner.

### Immunocytochemistry

iPSCs, neurons or astrocytes were seeded at 120,000 cells/well (24 well plate) on 12 mm coverslips precoated with Geltrex basement membrane matrix (A1413201, Thermo) and cultured as described above. Cells were fixed in 4% (w/v) formaldehyde/PBS for 15 minutes, permeabilized in 0.2% Triton X-100/PBS for 10 minutes at RT, blocked in 5% (v/v) FBS in PBS, and incubated with primary antibodies in 1% (v/v) FBS/PBS overnight at 4C. Following 3 washes in PBS, the cells were incubated for 1 hour with secondary antibodies (Alexa Fluor 488, 568, 647-conjugated; ThermoFisher). After 3 PBS washes, the coverslips were mounted, and the cells were imaged by confocal microscopy (Nikon CSU-W1-SORA).

### Live Imaging of Cellular Probes

For confocal microscopy of live iron probes, cells were plated at 120,000 cells/well in the center of MatTek glass bottom dishes (p35g-1.5–14-c) precoated with Geltrex basement membrane matrix (A1413201, Thermo) and cultured as described above. Cells were labeled with FerroOrange (Dojindo), HMRhoNox-M (Lumiprobe), Liperfluo (Thermo), LysoTracker Red (Invitrogen), CellROX (Thermo) and Hoechst according to the manufacturer’s specifications, and imaged on a Nikon CSU-W1 SoRa Spinning Disk confocal. CellLight Lamp1-emGFP BacMam 2.0 (Thermo) was used to visualize lysosomes. For high-content imaging of iron probes, iPSCs, neurons or astrocytes were plated at 10,000 cells per well in 96-well black-wall clear-bottom plates (Greiner). Live cells were labeled as above and imaged at 10x or 20x magnification, six fields per well, in the DAPI, GFP, RFP and Cy5 channels using the Lionheart FX high-content automated microscope (Agilent). Images were analyzed on the Gen5 software.

### Imaris analysis

Following confocal microscopy, the Imaris platform (Bitplane, Zürich, Switzerland) was used to analyze the localization and distribution of lysosomal iron in lysosomes. Z-stack confocal images of iAs were processed through the Imaris Surface Contour module to render lysosomal iron, lysosomes and nuclear staining to surfaces and measure the distance between lysosomes and proximity to the nuclear edge throughout z planes in the 3D volume.

### Statistical Analyses

All experiments were conducted at least three independent times for three differentiations. Error bars indicate mean +SD. Statistical analysis was performed using GraphPad Prism software, using a one-way ANOVA with Dunnett’s post-hoc or two-way ANOVA with Tukey’s post-hoc test.

## Results

### LRRK2 mutations dysregulate iron-related pathways in patient-derived iPSCs in a kinase-dependent manner

**Figure 1.**
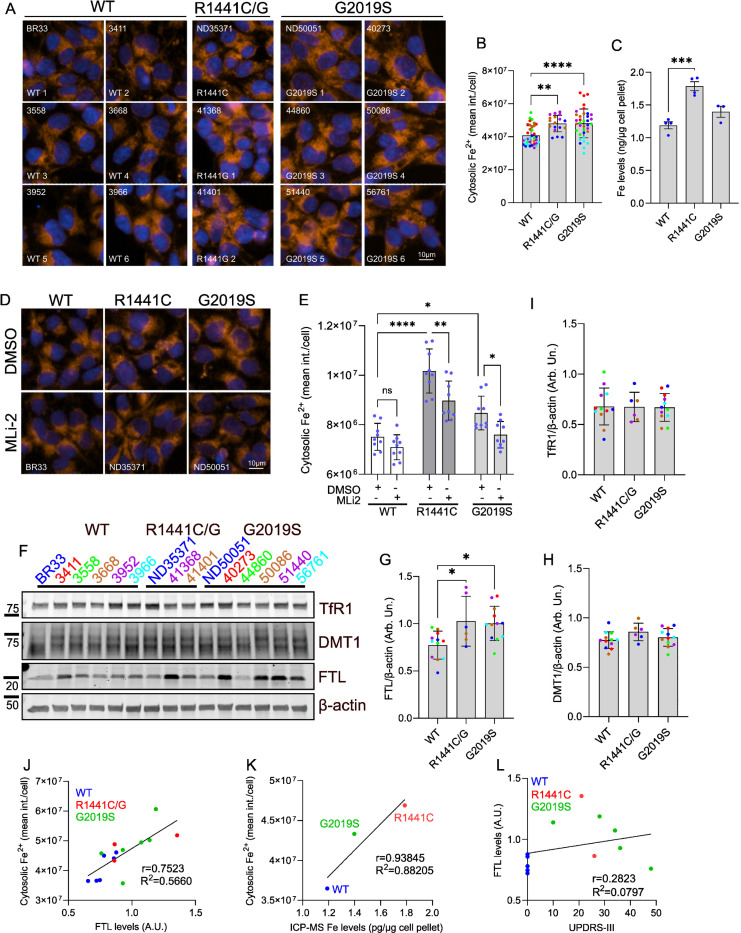
LRRK2 mutations dysregulate iron-related pathways in human iPSCs in a kinase-dependent manner. (A, B) Cytosolic free iron imaging and imaging (FerroOrange) in six WT, six G2019S, one R1441C, and two R1441G LRRK2 iPSC lines (PPMI, NINDS Repository) (N=6 wells each line; each line represented in a different color within each genotype group; one-way ANOVA, Dunnett’s post-hoc pair-wise comparison). (C) Quantitation of total iron in WT, R1441C and G2019S iPSCs (NINDS Repository) by ICP-MS. (D, E) Imaging and quantitation of cytosolic free iron (FerroOrange) in mutant LRRK2 iPSCs (NINDS Repository) following Mli-2 treatment (100μM, 7 days) (two-way ANOVA, Tukey’s post-hoc; N=9 biological replicates (>800 cells per N), Genotype ****p=0.0001, Treatment ****p<0.0001, Interaction p=0.2318). (F, G, H, I) Western blot and quantitation of levels of iron-related factors in WT, heterozygous R1441C/G, and G2019S iPSCs (N=2 biological replicates per line; 6 WT, 2 R1441G, 1 R1441C and 6 G2019S lines, one-way ANOVA, Dunnett’s post-hoc; *p<0.05). (J) Scatter plot showing a positive correlation between cytosolic iron (FerroOrange) and Ftl levels (r=0.7523). (K) Scatter plot showing a positive correlation between cytosolic iron (FerroOrange) and total iron levels by ICP-MS (r=0.93845; means of N>5 biological replicates shown per genotype). (L) Scatter plot showing no significant correlation between Ftl levels and clinical severity of patient donors (UPDRS-III).

In our previous work, we identified a signature of altered iron pathways in the kidneys of G2019S knockin mice by unbiased proteomics, highlighting divergent expression of endolysosomal and mitochondrial factors involved in iron regulation *in vivo*^31^. Separately, we reported increased accumulation of iron in the brains of homozygous G2019S LRRK2 mice following inflammation compared to WT controls^28^. These data were the first to suggest that G2019S LRRK2 dysregulates aspects of intracellular iron homeostasis in LRRK2 models. To examine whether these pathways are affected by endogenous LRRK2 mutations beyond G2019S, and when present in the heterozygous state associated with PD, we assessed different aspects of iron homeostasis in iPSCs from LRRK2 mutation carriers. We quanitified cytosolic iron levels in six WT, six G2019S, one R1441C, and two R1441G LRRK2 iPSC lines (all heterozygous) by high-content imaging of the FerroOrange probe, a fluorescent indicator of labile iron. We observed an increase of the steady state labile ferrous iron pool across different LRRK2 mutant lines compared to WT lines (Figure 1A, B). Each iPSC line is depicted in a different color to reflect the biological variability observed across the different patient-derived lines (Figure 1B). Upregulation of iron load in R1441C LRRK2 mutant iPSCs was further confirmed by ICP-MS (Figure 1C). Lastly, LRRK2 kinase inhibition reversed the increase in labile iron in heterozygous G2019S iPSC lines compared to WT with partial rescue on R1441C LRRK2 iPSCs (Figure 1D, E). Next, we investigated the levels of proteins involved in iron import (TfR1, DMT1) and iron storage (ferritin light chain; Ftl). We observed an overall increase in Ftl levels across the R1441C/G and G2019S mutant lines compared to WT iPSC controls (Figure 1F, G), while levels of TfR1 and DMT1 were comparable between lines (Figure 1F, H, I). Despite an overall effect of mutations on iron-related readouts, we noted biological variability in the levels of labile iron (Figure 1B) and ferritin (Figure 1G) across the different patient-derived iPSC lines. We noted moderate-to-strong correlation between labile iron levels (FerroOrange) and Ftl levels (R^2^=0.5660, F(1,13), p=0.0012) (Figure 1J), and a strong correlation between labile iron (FerroOrange) and total iron levels detected by ICP-MS (R2=0.8805. F(1,1), p=0.2247) (Figure 1K). Abnormal iron deposition in the SNc of PD patients is positively correlated to peripheral ferritin levels in the CSF and serum as well as motor symptom severity^54,55^. We did not observe a correlation between observed ferritin levels in the iPSC lines and motor symptom severity (UPDRS III scores) in LRRK2 PD or control groups reported for the iPSC donors (Figure 1L).

### LRRK2 mutations increase cellular iron in iPSC-derived neurons

**Figure 2.**
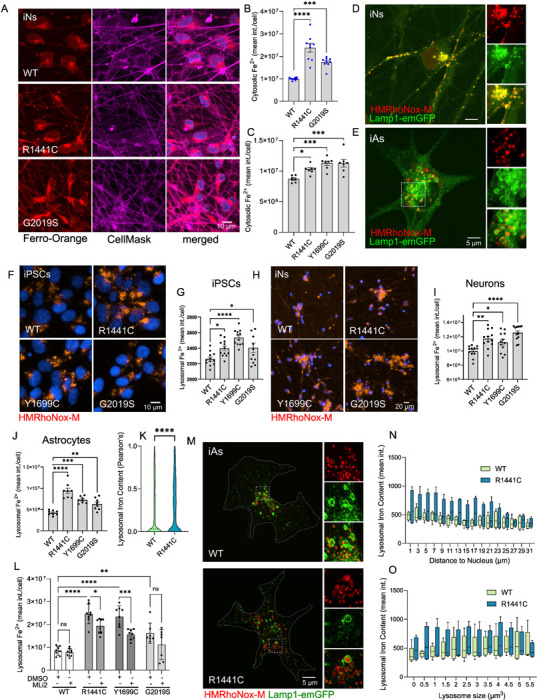
LRRK2 mutations increase lysosomal iron in isogenic neurons and astrocytes. Cytosolic iron imaging and quantitation in mature iNs carrying heterozygous LRRK2 mutations (A, B) as well as isogenic iNs versus controls (NIA, NIH) (C). Representative images of lysosomal iron (HMRhoNox-M) and Lamp1-emGFP by super-resolution confocal microscopy in (D) iNs and (E) iAs. Cytosolic free iron imaging and quantitation in heterozygous LRRK2 mutant isogenic iPSCs (F, G), isogenic iNs (H, I), and isogenic iAs (J, K) (one-way ANOVA, Dunnett’s post-hoc, N=8 biological replicates, (>800 cells per N), *p<0.05, **p<0.01, ***p<0.001, ****p<0.0001). (L) Lysosomal iron quantitation in iAs following treatment with MLi-2 in culture (100nM, 7 days) (two-way ANOVA, Tukey’s post-hoc; N=8 biological replicates (>800 cells per N), Genotype ****p=0.0001, Treatment ****p<0.0001, Interaction p=0.0844). (M) Super-resolution imaging of Lysosomal iron and Lamp1-emGFP in WT and isogenic R1441C iAs. Z-stack confocal images were 3D reconstructed in Imaris (Bitplane) and the frequency distribution of lysosomal iron content versus proximity to the nucleus (N) as well as lysosome size (O), were plotted.

Since iron dyshomeostasis is a key feature in PD pathology and our data support altered iron levels in LRRK2 mutant iPSCs, we next examined human iPSC-derived neurons to understand how this change translates in post-mitotic cells. iPSCs (NINDS Repository) were differentiated into mature cortical glutamatergic neurons (iNs) by forced expression of NGN2 via lentiviral gene delivery, according to published protocols^25,49,56^. We detected an increase in the labile iron pool across heterozygous R1441C and G2019S LRRK2 neurons compared to WT controls (Figure 2A, B). Considering the biological variability observed in these patient-derived iPSC lines (Figure 1), we next tested this phenotype in a series of isogenic heterozygous LRRK2 mutant iPSC lines. These lines were generated by CRISPR/Cas9 in a single female iPSC background, A18945, editing validated and subsequently characterized for morphology, karyotype abnormalities and differentiation potential^48^. NGN2-induced cortical neurons generated from the isogenic LRRK2 lines validated the phenotype with an increase in the labile iron pool across R1441C, Y1699C and G2019S LRRK2 compared to WT controls (Figure 2C), supporting an effect of diverse LRRK2 mutations on iron homeostasis.

### LRRK2 mutations cause lysosomal iron accumulation in neurons and astrocytes

While our data indicate changes in labile and overall cellular iron levels, we did not initially assess subcellular distribution of iron with the FerroOrange iron probe. This probe detects free ferrous iron in the cytosol as well as in different organelles including ER, mitochondria and endolysosomes^57–59^ (Supplementary Figure 1). Given the role of lysosomes as sites of cellular iron storage regulating intracellular iron availability^60–62^, as well as studies by us and others demonstrating effects of LRRK2 mutations on lysosome-related pathways^24,25,28,63–65^, we initially focused on evaluating perturbation of lysosomal iron levels in LRRK2 mutant cells.

We employed an iron probe (HMRhoNox-M) that specifically detects ferrous iron (Fe^2+^) in lysosomes and assessed lysosomal iron load across iPSCs, cortical NGN2-induced neurons (iNs) and induced-astrocytes (iAs)^52^ by high-content imaging and super-resolution microscopy (Figure 2E to O). iAs were generated by viral induction of SOX9 and NFIB expression^52^, and expression of astrocytic markers and LRRK2 was confirmed at ~21 days *in vitro* (Supplementary Figure 2). Initially, we tested specificity of HMRhoNox-M to lysosomes by comparing HMRhoNox-M staining to Lamp1-emGFP localization (Figure 2D, E). In both iPSC-derived human neurons (Figure 2D) and astrocytes (Figure 2E), HMRhoNox-M showed exclusive localization within Lamp1-emGFP positive structures, validating specificity to the lysosomal compartment. Assaying lysosomal iron levels in iPSCs carrying heterozygous LRRK2 mutations, revealed an increase in R1441C, Y1699C and G2019S LRRK2 mutant lines compared to isogenic WT controls (Figure 2F, G). This LRRK2 mutation-driven increase in lysosomal iron load was conserved in mature iNs (Figure 2H, I) and astrocytes (Figure 2J, K) generated from these isogenic lines. To test whether lysosomal iron increase in LRRK2 mutant iAs is dependent on LRRK2 kinase activity, iAs were treated with MLi-2 for 7 days prior to analysis (Figure 2L). Inhibition of LRRK2 kinase activity reduced lysosomal iron levels in all mutant lines but not WT, with partial rescue in the R1441C and Y1699C lines and complete rescue in the G2019S line (Figure 2L). LRRK2 is active on the lysosomal membrane with studies suggesting that lysosomal positioning modulates LRRK2 activity towards Rab GTPases^66^. We therefore assessed the distribution of iron content in lysosomes in relation to distance from the nucleus in mature iAs. We found that the increase in lysosomal iron content with the R1441C mutation was more prominent in perinuclear compared to peripheral lysosomes and also primarily observed in smaller (< 3 μm^3^) lysosomes (Figure 2 M, N, O). These data are consistent with perinuclear lysosomes being sites of active LRRK2 signaling^66^, as demonstrated by our work and that of others showing LRRK2-dependent effects on their function and proteolytic activity^22,49,67,68^. Together these results provide evidence for altered lysosomal ferrous iron levels associated with LRRK2 mutations.

### Ferritin levels are dysregulated in isogenic LRRK2 mutant iPSCs

**Figure 3.**
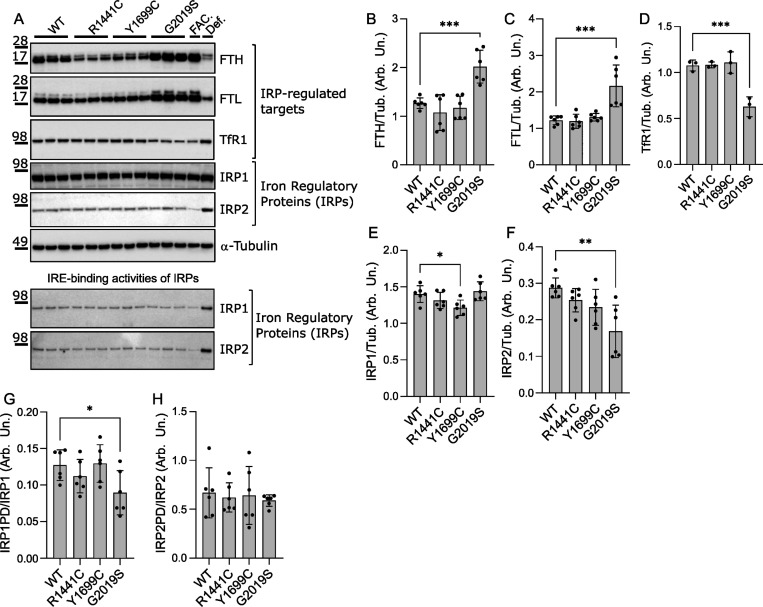
Ferritin heavy chain is dysregulated in isogenic LRRK2 mutant iPSCs. (A-F) Western blot analysis of IRPs (IRP1, IRP2) and IRP-regulated proteins (Fth1, Ftl, TfR1) in isogenic LRRK2 mutant iPSCs. (A bottom panel, G, H) Western blot of IRE-bound IRP1 and IRP2 in isogenic LRRK2 mutant iPSCs and quantitation of IRE-binding. (one-way ANOVA, Dunnett’s post-hoc, N=6 biological replicates, *p<0.05, **p<0.01, ***p<0.001).

Altered cytosolic and lysosomal iron demonstrate an impairment in how cells regulate their iron levels and distribution, so we further evaluated expression of key proteins involved in these processes. Iron uptake, transport, and storage are finely regulated at the post-transcriptional level by the iron regulatory protein (IRP) and iron-responsive element (IRE) signaling pathways^69^. IRP1 and IRP2 are RNA-binding proteins that interact with IREs located within the untranslated regions of specific mRNAs, to modulate iron homeostasis. These interactions repress ferritin (both Fth1 and Ftl) and Fpn mRNA translation while stabilizing TfR mRNA^70–72^. In proliferating iPSCs cultured without iron supplements, we observed an increase in total Fth1 and Ftl levels in G2019S LRRK2 cells compared to isogenic WT controls, while R1441C LRRK2 iPSCs showed a trend of lower Fth1 (Figure 3A, B, C). Consistent with an IRP1-mediated increase in ferritin protein levels, G2019S iPSCs also had lower basal TfR1 levels as compared to WT (Figure 3A, D). We therefore directly measured IRP1 and IRP2 levels and activity in these LRRK2 mutants. We observed a trend of downregulation of IRP1 in the ROC-COR domain mutants that reached significance in Y1699C iPSCs, supporting impairment in IRP1 signaling (Figure 3A, E). Separately, the G2019S iPSCs showed lower IRP2 expression levels with the ROC-COR domain mutants showing a trend toward decrease (Figure 3F). To evaluate IRE-binding activities of IRP1 and IRP2, we used a biotinylated-IRE probe to separate IRE-binding IRPs from cell lysates and analyzed by immunoblotting, as before^53^. The IRE-binding activity of IRP1 was lower in G2019S iPSCs compared to the isogenic WT controls while IRP2 activity showed a high degree of variability in these assays which precluded any determination (Figure 3G, H).

IRP1 activity is regulated by intracellular iron levels but can also be influenced by Fe-S cluster biogenesis as it requires a 4Fe-4S cluster to function as cytosolic aconitase^70,73^ and loss of Fe-S clusters shifts IRP1 to its IRE-binding form, mimicking iron deficiency. Similarly, IRP2, though not Fe-S dependent, is stabilized when cytosolic iron is low, which can also happen if Fe-S cluster biogenesis is impaired in mitochondria. To evaluate a potential effect of iron dyshomeostasis on mitochondrial function, we assayed levels of mitochondrial Fe-S-containing subunits (Supplementary Fig 3), noting no differences in basal levels of MTCO1, UQCRFS1, UQCRC1, POLD1 and SDHB. While our data suggest a mild effect of LRRK2 mutations on IRP activity, we observe significant effects on ferritin levels, suggesting that separate mechanisms on ferritin homeostasis at the protein level may be at play.

### Rab8a deficiency phenocopies LRRK2 mutation-driven iron accumulation

**Figure 4.**
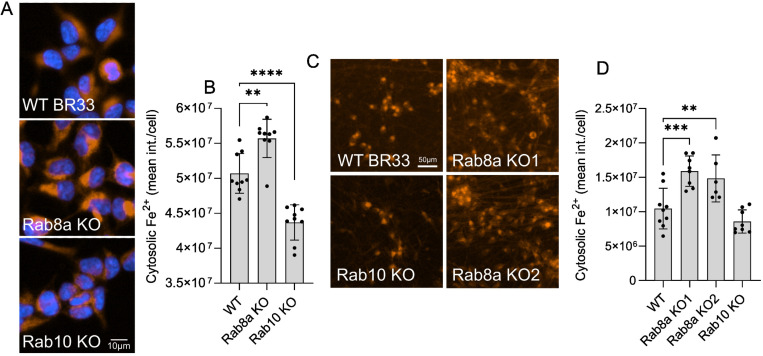
Rab8a deficiency phenocopies LRRK2 mutation-driven iron accumulation. (A, B) Imaging of cytosolic free iron and quantitation of iron levels in WT, Rab8a KO and Rab10 KO iPSCs by high-content imaging. (C, D) Imaging and quantitation of free cytosolic iron in Rab8a KO and Rab10 KO iNs compared to isogenic controls. (one-way ANOVA, Dunnett’s post-hoc, N=6 biological replicates, *p<0.05, **p<0.01, ***p<0.001).

Phosphorylation of Rab GTPases by LRRK2 locks the molecules in an inactive state affecting their function^28,32,49,74^. Recently, we showed that LRRK2-driven phosphorylation of Rab8a impairs its function in TfR trafficking, linking LRRK2 to iron homeostasis^28^. Furthermore, we demonstrated that Rab8a KO and Rab10 KO neurons show distinct phenotypes in lysosomal integrity and PD-pathology related proteostasis^49^. To investigate whether inactivation of Rab8a uniquely drives dysregulation of cellular iron levels, we used CRISPR/Cas9 gene editing to generate Rab8a KO and Rab10 KO iPSCs^49^ and HEK293 cells (Supplementary Figure 4), with matched isogenic controls and assayed cellular iron load as before using the FerroOrange probe. Rab8a KO iPSCs showed increased labile iron compared to isogenic WT controls, mimicking mutant LRRK2, while the opposite effect was seen in Rab10 iPSCs (Figure 4A, B). These data were replicated in iNs, with Rab8a deficiency resulting in accumulation of free cellular iron while this was not mirrored by Rab10 KO iNs (Figure 4C, D). These findings provide additional support to the hypothesis that reduced Rab8a activity is at least in part responsible for LRRK2-dependent changes in cellular iron storage.

**Figure 5.**
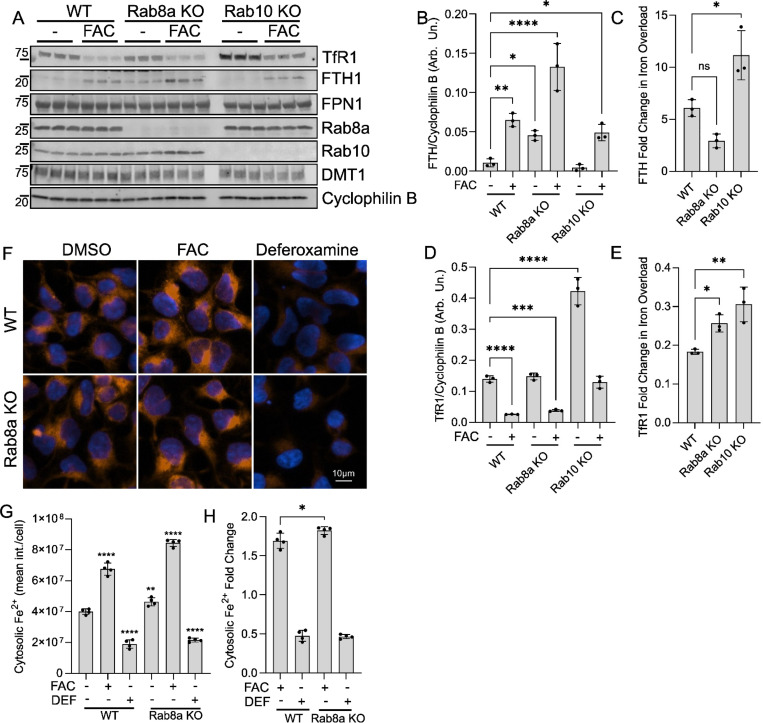
Rab8a deficiency impairs ferritin heavy chain regulation in HEK293 cells. (A-C) Western blot analysis of iron-related proteins in Rab8a KO and Rab10 KO HEK293 cells in basal and iron overload (FAC) conditions (two-way ANOVA, Tukey’s post-hoc; N=3 biological replicates; Fth1: Genotype ****p=0.0001, Treatment ****p<0.0001, Interaction p=0.0508; TfR1: Genotype ****p=0.0001, Treatment ****p<0.0001, Interaction ****p<0.0001). (F-H) Imaging and quantitation of free iron in Rab8a KO HEK293 cells under iron overload (FAC) and iron chelation (DEF) conditions. (one-way ANOVA, Dunnett’s post-hoc, N=4 biological replicates, *p<0.05, **p<0.01, ***p<0.001).

To investigate how Rab8a and Rab10 deficiency affect cellular iron homeostasis, we assayed levels of iron-related proteins at basal conditions, and in conditions of iron overload following overnight incubation in ferric ammonium citrate^75^. We observed an increase in basal Fth1 levels in Rab8a KO HEK293T cells compared to WT or Rab10 KO cells (Figure 5A, B). In conditions of iron overload, Rab8a cells showed a blunted increase in Fth1 expression compared to Rab10 KO cells, which showed the highest fold increase compared to WT (Figure 5A, C). Basal TfR1 levels were increased in Rab10 KO cells while both Rab KO cell lines showed higher fold decrease in TfR1 levels in iron overload conditions compared to WT (Figure 5A, D, E). Rab8a cells accumulated higher cellular iron levels compared to isogenic WT controls, in exogenous iron overload conditions, while this was ameliorated by iron chelation (Figure 5F, G, H). Taken together, these findings support that Rab8a inactivation mirrors the iron dyshomeostasis seen in LRRK2 mutations, marked by elevated free iron and blunted ferritin upregulation, whereas Rab10 deficiency appears to engage distinct, potentially compensatory trafficking pathways that differentially modulate iron homeostasis.

### Iron chelation protects against LRRK2-driven ROS accumulation and ferroptosis

**Figure 6.**
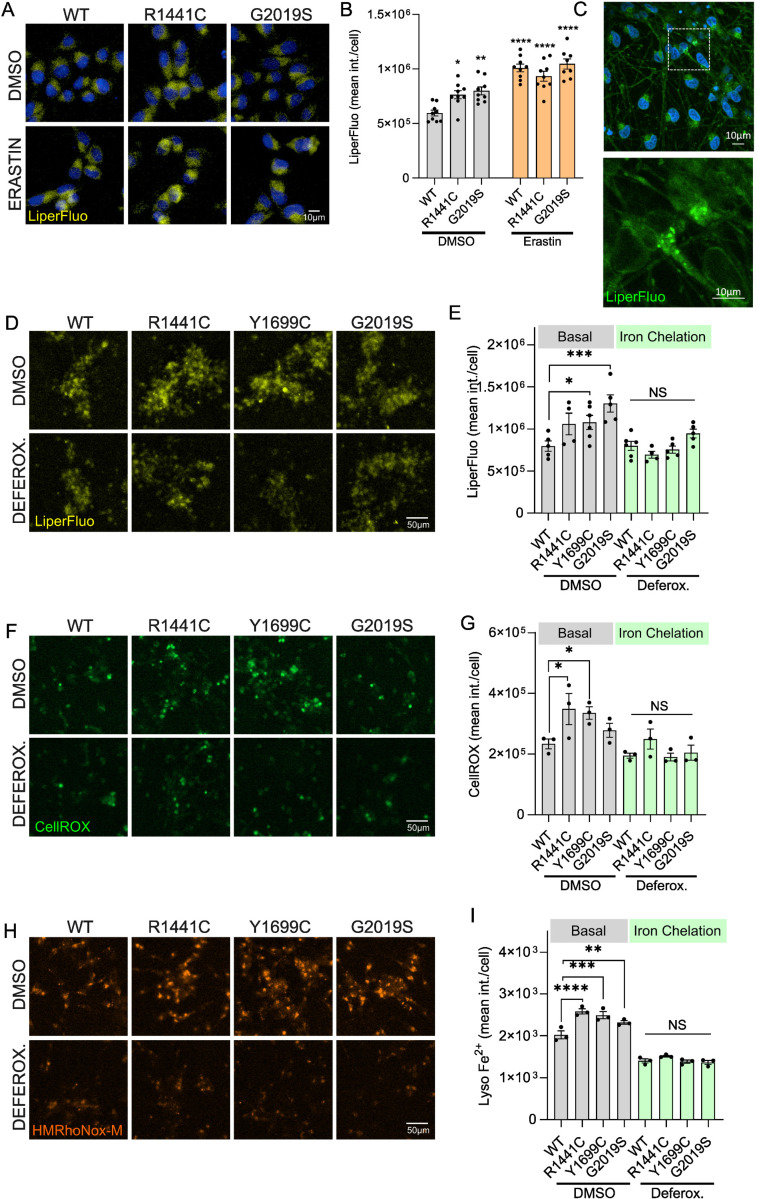
Iron chelation protects against LRRK2-driven ROS accumulation and ferroptosis. (A) Isogenic iPSCs carrying LRRK2 mutations stained with Liperfluo to detect lipid peroxidation following treatment with Erastin or vehicle. (B) Quantitation of Liperfluo intensity by high-content imaging (two-way ANOVA, Tukey’s post-hoc; N=9 biological replicates (>800 cells per N), Genotype *p=0.0105, Treatment ****p<0.0001, Interaction **p=0.0072). (C) Representative confocal image of iNs stained with Liperfluo. (D, E) Lipid peroxidation imaging and quantitation in iNs carrying heterozygous LRRK2 mutations versus isogenic WT control, following treatment with Deferoxamine. (F, G) High-content imaging and analysis of CellROX dye in LRRK2 mutant iNs. (H, I) High-content imaging and analysis of lysosomal iron content (HMRhoNox-M). (Liperfluo: two-way ANOVA, Tukey post-hoc; N=3 biological replicates (>800 cells per N), Genotype ***p=0.0009, Treatment ****p<0.0001, Interaction *p=0.0497; CellROX: two-way ANOVA, Tukey post-hoc; N=3 biological replicates (>800 cells per N), Genotype *p=0.02, Treatment ***p=0.0002, Interaction p=0.3923; HMRhoNox-M: two-way ANOVA, Tukey post-hoc; N=3 biological replicates (>800 cells per N), Genotype ***p=0.0003, Treatment ****p<0.0001, Interaction **p=0.0034).

Ferroptosis, the process of iron-dependent lipid peroxidation and cell death, is an emerging pathogenetic mechanism of interest in the neurodegeneration in PD^16,75–77^. Iron catalyzes the conversion of hydrogen peroxide to ROS via the Fenton reaction, resulting in oxidative injury. Ferroptosis in neurodegeneration involves the simultaneous accumulation of brain iron and lipid peroxidation which trigger a cascade of pathologic events including inflammation, myelin sheath degeneration, glial dysregulation and cell death^78^. LRRK2 mutations have been linked to increased ROS in different model systems but how LRRK2 signaling leads to increased cell stress remains largely unclear^79,80^. To test whether heterozygous LRRK2 mutations result in ROS-induced damage in our systems, we employed Liperfluo, a live cell imaging probe that detects lipid peroxide-specific oxidation^81^. At basal conditions in proliferating cells, we detected an increase in lipid peroxidation in R1441C and G2019S iPSCs compared to isogenic WT controls (Figure 6A, B). These data are in line with recent work that places LRRK2 in a ferroptosis pathway and highlighting lipid peroxidation relevant to LRRK2 signaling^82,83^. Erastin is a small molecule ferroptosis inducer that inhibits the cystine/glutamate antiporter leading to depletion of glutathione and accumulation of lipid peroxides and cell damage^84–87^. When challenged with erastin acutely, all lines showed increased lipid peroxidation, as expected (Figure 6B). There were no LRRK2-dependent differences following this exogenous stress. To investigate whether a similar LRRK2-driven phenotype is observed in neurons, mature iNs were stained with Liperfluo and analyzed by confocal microscopy and high-content imaging (Figure 6C, D, E). LRRK2 genotype had a significant effect on lipid peroxidation levels in heterozygous LRRK2 mutant iNs (two-way ANOVA) with an increase in Y1699C and G2019S LRRK2 lines compared to WT (Figure 6D, E). To test whether this LRRK2-driven phenotype is dependent on cellular iron, iNs were treated with deferoxamine overnight prior to imaging. Iron chelation by deferoxamine rescued the increase in lipid peroxidation seen with mutant LRRK2 (Figure 6D, E). Assessing cellular ROS levels by CellROX imaging revealed a genotype-driven accumulation across mutations that was likewise rescued by iron chelation (Figure 6F, G). Lastly, intralysosomal iron chelation was confirmed by imaging that revealed a decrease in lysosomal iron load across WT and mutant iNs following treatment with deferoxamine (Figure 6H, I). These data support an effect of endogenous heterozygous LRRK2 mutations on neuronal ROS and lipid peroxidation that is rescued by iron chelation.

## Discussion

The pathological deposition of iron in the PD brain has been widely documented for over three decades, with early histochemical studies identifying iron accumulation in the substantia nigra of post-mortem PD tissue^88,89^, and subsequent MRI and spectroscopic studies confirming region-specific iron overload *in vivo*^9,90,91^. However, the mechanisms by which genetic risk factors may contribute to intracellular iron mismanagement have remained poorly defined. In this study we show that PD-linked LRRK2 mutations disrupt iron handling in human iPSCs, neurons, and astrocytes, with a striking and convergent increase in lysosomal ferrous iron. This iron accumulation is kinase-dependent and reversible, implicating chronic aberrant LRRK2 signaling in the misregulation of vesicular iron storage. By integrating imaging, biochemical assays, and isogenic models, we report that Rab8a deficiency phenocopies LRRK2-driven iron defects, and demonstrate that LRRK2 mutations sensitize neurons to iron-driven oxidative stress and ferroptosis. These findings provide a mechanistic link between LRRK2 activity and iron toxicity, offering new insight into the cell-autonomous vulnerabilities that may drive neurodegeneration in PD.

Most pathogenic LRRK2 mutations cluster within the kinase and the Roc-COR tandem domains of the protein, and augment the kinase activity of LRRK2 towards a subset of Rab GTPases^30^. Rab GTPases control many aspects of intracellular vesicle trafficking by acting as regulatable switches that recruit effector molecules to distinct intracellular membranes^32,92^. We and others, have shown that phosphorylation of Rab GTPase by mutant LRRK2, including Rab8a and Rab10, interferes with their function and alters endolysosomal dynamics^22,24,28,30,31,93–95^. Furthermore, it has been reported that recruitment of LRRK2 to damaged lysosomes along with Rab8a and Rab10 and effects on lysosomal integrity^24,28,63(p10)^. Studies have shown divergent effects of Rab8a and Rab10 in cilliogenesis^96^ and we recently reported distinct lysosomal phenotypes between Rab8a KO and Rab10 KO cells^49^. Here, we extend this work by demonstrating that Rab8a, but not Rab10, loss partially mimics aspects of iron impairment observed in LRRK2 mutant cells, including upregulation of labile cellular iron and ferritin. These results highlight a functional divergence between Rab8a and Rab10 in iron handling and point to Rab8a as a likely mediator of LRRK2-dependent iron misregulation, potentially through its role in transferrin receptor trafficking and lysosomal function^28,36^.

Lysosomes serve as crucial hubs for iron storage and availability^61,62^. Physiologic delivery of iron to cells is primarily mediated by transferrin, which binds ferric iron in the extracellular environment and is internalized through clathrin-dependent endocytosis via the transferrin receptor, while non-transferrin bound iron (NTBI) uptake can play a role in iron uptake associated with systemic iron overload or trauma^97^. Within endolysosomes, the acidic environment facilitates the release of iron from transferrin and endolysosomal ferrireductases reduce Fe^3+^ to Fe^2+^, allowing iron exit into the cytoplasm through DMT1. This endolysosomal acidification step is critical for iron release and trafficking, and its disruption has been linked to cellular iron deficiency, mitochondrial dysfunction, and inflammation^62,98,99^. In fact, inhibition of the lysosomal v-ATPase triggers cellular iron dyshomeostasis, resulting in impaired mitochondrial function and non-apoptotic cell death *in vivo*^98^. Here, by using a lysosomal-specific iron probe we found that LRRK2 mutations lead to increased lysosomal ferrous iron content, an effect observed in both terminally differentiated neurons and astrocytes. These data, together with previous reports linking LRRK2 to lysosomal function^24,28,63^, and specifically intralumenal pH^22,68^, suggest that LRRK2 mutations disrupt normal lysosomal iron handling by altering lysosomal homeostasis. We found that perinuclear lysosomes have a pronounced increase in ferrous iron in LRRK2 mutant astrocytes compared to peripheral lysosomes. Studies have shown that perinuclear lysosomes have lower luminal pH and higher protease activity compared to distal ones^67^. Lysosomal positioning provides regulation of LRRK2 activity towards Rab GTPases with perinuclear lysosomes harboring enhanced kinase-driven Rab signaling^66^. We and others have reported dysregulation of lysosomal acidification by mutant LRRK2 in different cellular models^22,27,68,100,101^. It is plausible that impairment of endolysosomal iron reduction may be altering cellular iron content and availability in the context of LRRK2 mutations.

Iron-induced oxidative stress is a well-documented contributor to neurodegeneration, and our results support a role for LRRK2 mutations in exacerbating this process. Iron-induced cell death, termed ferroptosis, has emerged as a key mechanism contributing to oxidative damage and neuronal loss in PD models^16,75–77^. The convergence of aberrant brain iron and lipid peroxidation initiates downstream pathology marked by inflammation, glial dysfunction, demyelination, and ultimately neuronal death^78^. Here, we observed increased lipid peroxidation and ROS levels in mutant LRRK2 neurons, which were reversed by iron chelation. LRRK2 has been linked to ROS accumulation in different cells models^79,80^, and a recent study reported an effect of LRRK2 mutations in NOX2 activity that in turn has been linked to ferroptosis^80,102,103^. It is plausible that dysregulation of the labile and lysosomal iron pools by LRRK2 mutations contribute to the reported effects of LRRK2 on cellular ROS. Lysosomal function is key to iron homeostasis and cell health. The lysosomal membrane is exposed to redox-active iron and is an initial target of intracellular oxidant damage, with studies reporting a protective role for intralysosomal iron chelation to ROS damage^104^. A recent study identified the lysosomal protein prosaposin as an important regulator of lipid peroxidation and neuronal ROS^81^. LRRK2 signaling has been linked to prosaposin function *in vitro* and *in vivo*, highlighting a potential functional interaction that could mediate ferroptosis signaling in this context^105,106^. What remains to be established is whether dysregulation of lysosomal iron storage by mutant LRRK2 directly drives cellular ROS damage, or whether aberrant transport of endolysosomal iron to the labile or mitochondrial iron pools contributes pathologic effects. Given that deferoxamine attenuated both ROS and lysosomal iron content further strengthens the connection between lysosomal iron mismanagement and oxidative stress in PD-relevant cell types. Ferroptosis inhibitors have shown promise in mitigating neurodegeneration in cell and *in vivo* preclinical models^16,78,107^, thus targeting lysosomal LRRK2-mediated iron dysregulation may represent a viable therapeutic strategy.

Cellular iron homeostasis is tightly regulated at the post-transcriptional level by IRPs, which bind to IREs in target mRNAs to control the expression of key iron-handling proteins^69,70,72,73^. In our models, ferritin levels were dysregulated in G2019S LRRK2 lines, while IRP1/2 activity showed genotype-specific variation. Notably, cytosolic ferritin levels did not correlate strictly with IRP binding activity, implying that lysosomal dysfunction and impaired ferritin turnover may dominate ferritin regulation in this context. Interestingly, G2019S showed stronger effects on IRP/ferritin regulation compared to R1441C and Y1699C, suggesting that distinct LRRK2 mutations may impact iron homeostasis via partially divergent mechanisms. Disruption of ferritinophagy, a lysosome-mediated pathway that regulates ferritin turnover and iron release^19,108^, may underlie the elevated ferritin levels and lysosomal iron accumulation we observe in LRRK2 mutant cells, suggesting that impaired ferritin degradation contributes to ferroptotic vulnerability via dysregulated iron recycling.

Our data reveal that LRRK2 mutations drive lysosomal iron accumulation in both neurons and astrocytes, supporting a model in which iron dysregulation arises through both cell-autonomous and non-cell-autonomous mechanisms. Astrocytes are essential regulators of brain iron homeostasis, not only sequestering and buffering excess iron, but also modulating iron availability at synaptic interfaces by secreting factors that support neuronal iron uptake^37,38,41–43,109^. By shaping the extracellular iron landscape, astrocytes maintain appropriately low synaptic levels and influence neuronal redox balance^41,109^. Astrocytes are relevant to LRRK2 biology as they express high levels of LRRK2^27,110,111^, and their cytokine profile is modulated by LRRK2 kinase activity^39,40,44^. The increase in lysosomal ferrous iron observed in LRRK2 mutant astrocytes suggests a failure of astrocytic iron buffering capacity, that in an *in vivo* setting would translate to alterations of the synaptic iron microenvironment. In parallel, the accumulation of lysosomal iron in LRRK2 mutant neurons may reflect intrinsic defects in iron trafficking and storage, further sensitizing neurons to oxidative injury and ferroptosis. These findings highlight the dual contribution of LRRK2 to neurodegeneration: through direct, cell-autonomous disruption of neuronal iron handling, and indirectly by impairing astrocyte-mediated regulation of extracellular iron homeostasis.

Based on our current findings from human iPSC-derived models, further work will be needed to confirm whether similar lysosomal iron redistribution occurs *in vivo* in preclinical models where we previously reported effects on global iron pathways^28,31^. Additionally, while iron chelation and LRRK2 inhibition both rescued aspects of the phenotype, a dual therapeutic strategy remains to be tested in preclinical models. Importantly, iron chelation is an effective treatment in Neurodegeneration with Brain Iron Accumulation that often includes parkinsonism symptoms^112–115^, but recent clinical trials in PD failed^116^ suggesting that targeting precise iron pathways in the brain rather than systemic chelation is a promising avenue for altering disease progression

## Conclusion

Our results position iron dysregulation as a central node in the pathogenic cascade downstream of LRRK2 mutations. We propose that LRRK2 functions as a key regulator of neuronal and glial iron homeostasis through its effects on lysosomal iron trafficking and that disruption of this function results in redox imbalance and ferroptotic vulnerability. Combined with our findings on the LRRK2 substrate Rab8a, this work offers new mechanistic insight into PD pathogenesis and providing a rational basis for combinatorial therapeutic strategies. Together, these data offer a framework for understanding how genetic PD risk converges on iron homeostasis and highlight new pathogenic pathways and opportunities for therapeutic intervention.

## Supplementary Material

Supplement 1

## Data Availability

Materials and additional details can be made available by the corresponding authors upon request.

## List of abbreviations

PD: Parkinson’s disease
iPSC: Induced pluripotent stem cell
iNs: Induced neurons
iAs: Induced astrocytes
LRRK2: Leucine-rich repeat kinase 2
ROS: Reactive oxygen species
FAC: Ferric ammonium citrate
DEF: Deferoxamine
TfR1: Transferrin receptor 1
DMT1: Divalent metal transporter 1
Fth1: Ferritin heavy chain
Ftl: Ferritin light chain
IRP: Iron regulatory protein
IRE: Iron-responsive element
ICP-MS: Inductively coupled plasma mass spectrometry
QSM: Quantitative susceptibility mapping
PPMI: Parkinson’s Progression Markers Initiative
ANOVA: Analysis of variance
WT: Wild-type
