# Supplementary material for: Parkinson’s disease LRRK2 mutations dysregulate iron homeostasis and promote oxidative stress and ferroptosis in human neurons and astrocytes
Source: bioRxiv. 2025 Sep 28:2025.09.26.678370. Preprint. [Version 1] doi: 10.1101/2025.09.26.678370 (PMC12802402; doi:10.1101/2025.09.26.678370)
Supplement: Supplement 1 [file media-1.pdf]

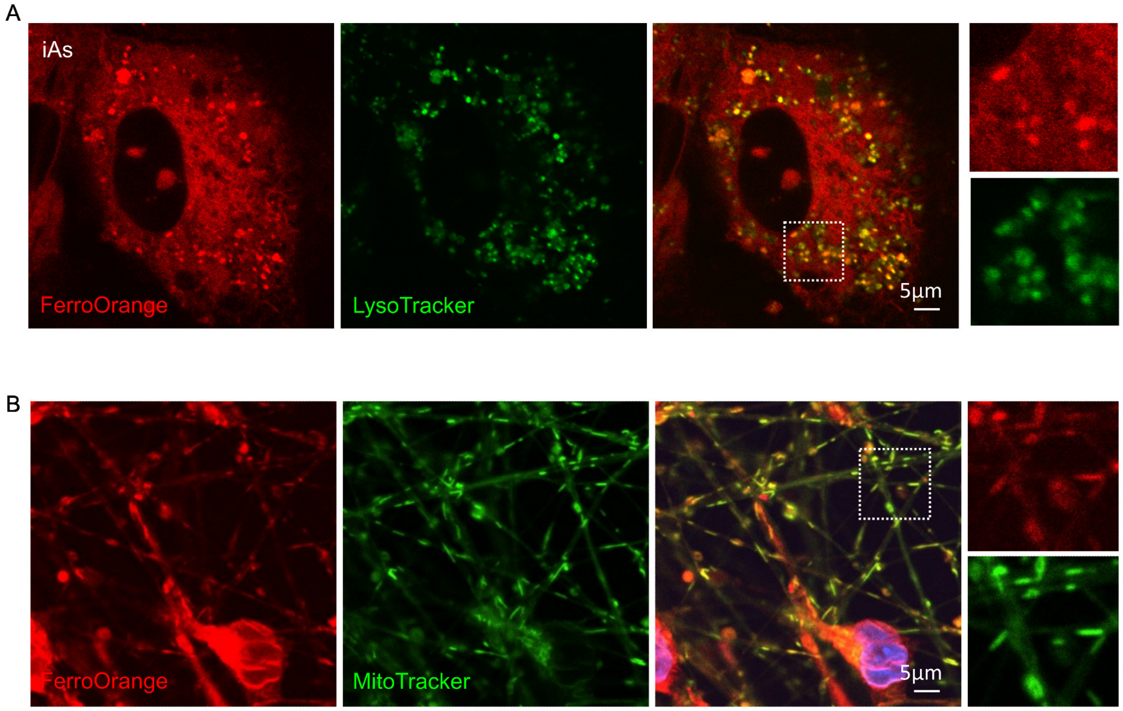

Supplementary Figure 1. Live imaging of FerroOrange iron probe. iAs (A) and iNs (B) were stained with the FerroOrange iron probe and LysoTracker or MitoTracker dyes, respectively, and imaged by confocal microscopy.

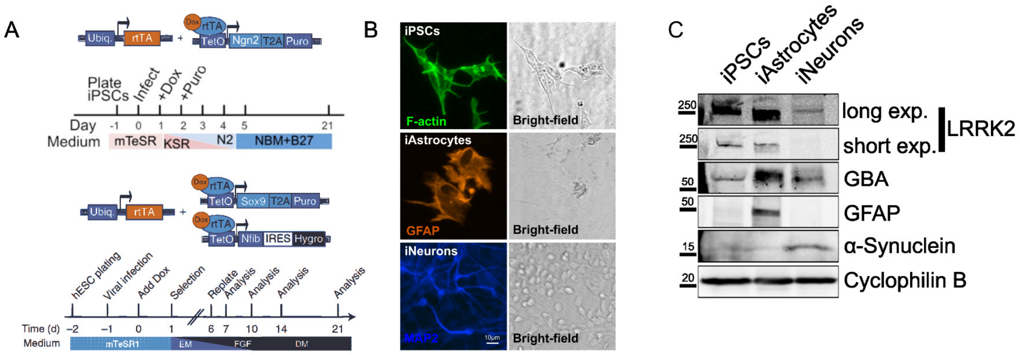

Supplementary Figure 2. iN and iA differentiation protocols and validation of neuronal and astrocytic markers. (A) iPSCs were differentiated into iNs by forced expression of Ngn2, and into iAs by forced expression of Sox9 and Nfib, according to published protocols. (B, C) Expression of MAP2, GFAP and α-synuclein was validated by ICC and immunoblotting.

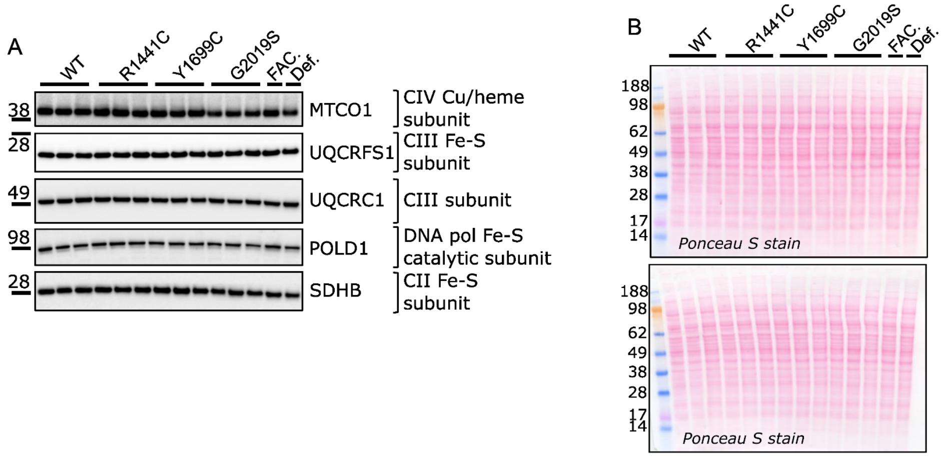

Supplementary Figure 3. Expression of Fe-S cluster containing proteins in iPSCs. (A) Isogenic iPSCs carrying LRRK2 mutations were analyzed for expression of Fe-S containing factors by immunoblot. (B) Equal loading was further validated by ponceau staining.

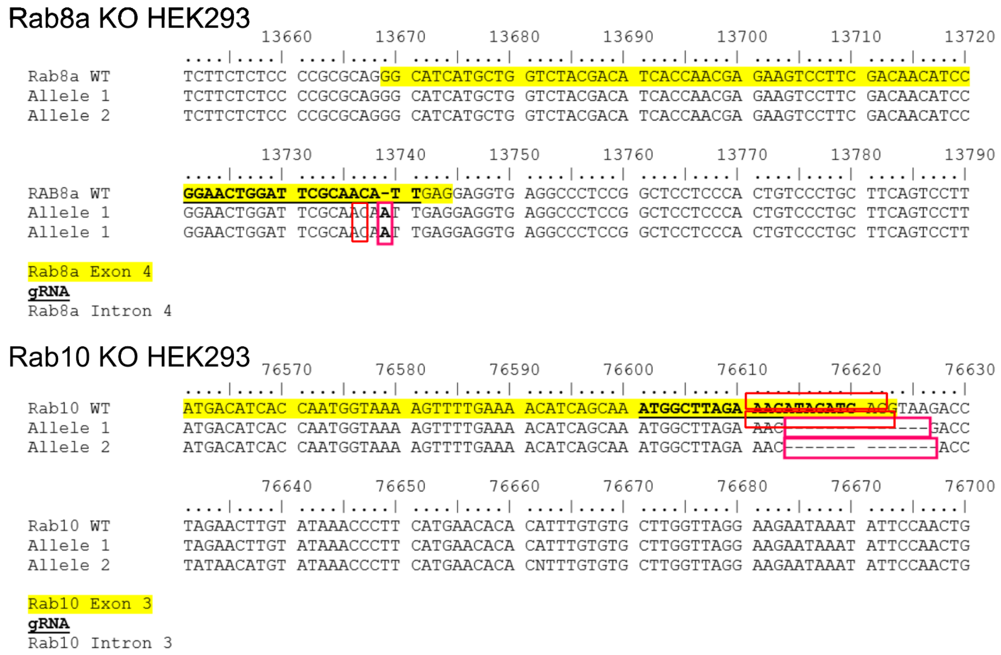

Supplementary Figure 4. Validation of *RAB8a* KO and *RAB10* KO HEK293 clones by Sanger sequencing. HEK293 Clones of each *RAB* GTPase edited line were validated by Sanger sequencing and used for further experimentation.

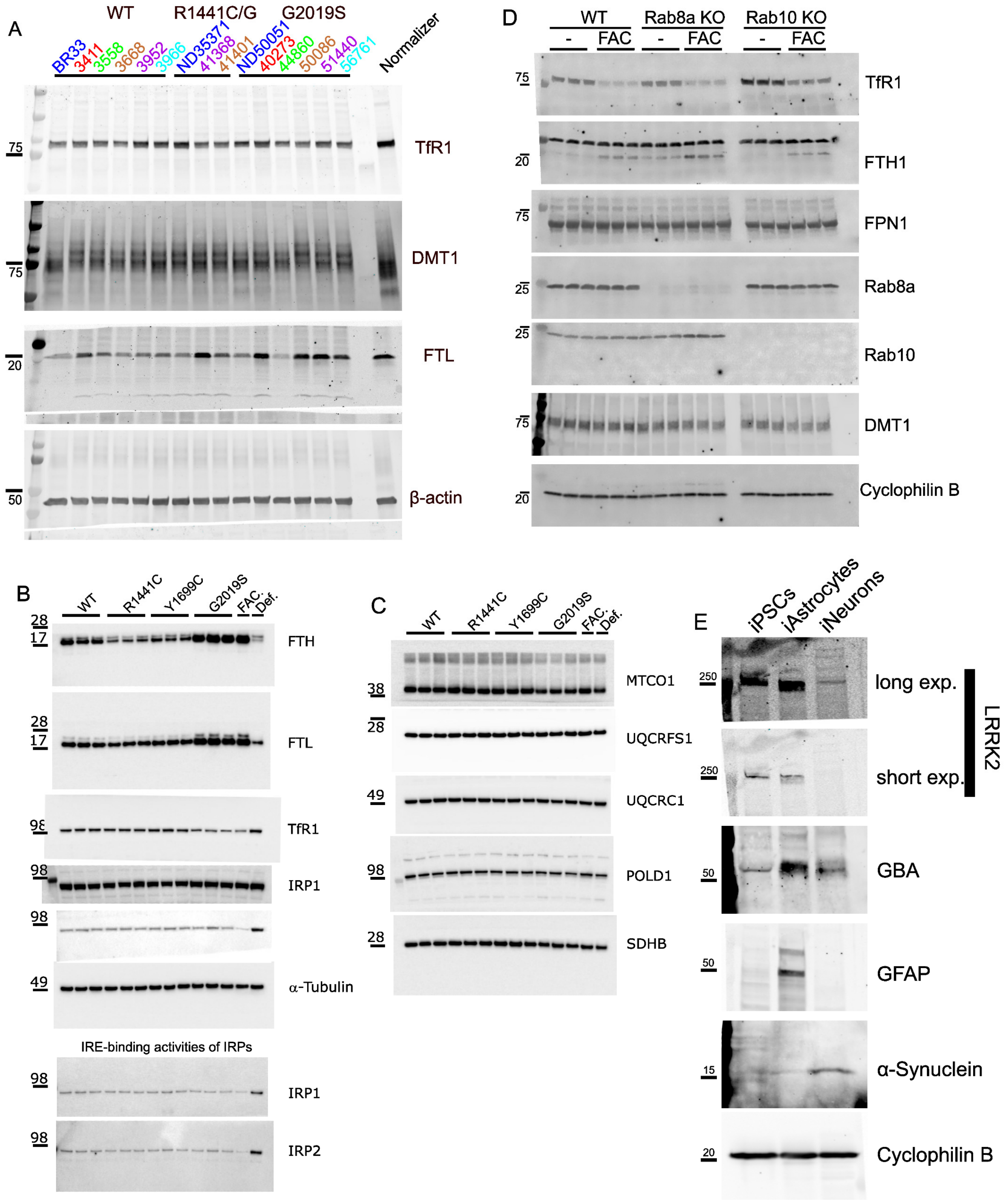

Supplementary Figure 5. Uncropped blots. Full blots are presented from: (A) Figure 1F, (B) Figure 3A, (C) Suppl. Figure 2C, (D) Suppl. Figure 3A.
